# Pristine and Magnetic Kenaf Fiber Biochar for Cd^2+^ Adsorption from Aqueous Solution

**DOI:** 10.3390/ijerph18157949

**Published:** 2021-07-27

**Authors:** Anwar Ameen Hezam Saeed, Noorfidza Yub Harun, Suriati Sufian, Muhammad Roil Bilad, Zaki Yamani Zakaria, Ahmad Hussaini Jagaba, Aiban Abdulhakim Saeed Ghaleb, Haetham G. Mohammed

**Affiliations:** 1 Department of Chemical Engineering, University Teknologi PETRONAS, Seri Iskandar 31750, Malaysia; anwar_17006829@utp.edu.my (A.A.H.S.); suriati@utp.edu.my (S.S.); 2 Centre of Urban Resource Sustainability, University Teknologi PETRONAS, Bandar Seri Iskandar 32610, Malaysia; 3 Faculty of Integrated Technologies, University Brunei Darussalam, Jalan Tungku Link, Gadong BE1410, Brunei; roil.bilad@ubd.edu.bn; 4 School of Chemical & Energy Engineering, University Teknologi Malaysia, Skudai 81310, Malaysia; zakiyamani@utm.my; 5 Department of Civil and Environmental Engineering, University Teknologi PETRONAS, Bandar Seri Iskandar 32610, Malaysia; ahjagaba@gmail.com (A.H.J.); aiban_17004546@utp.edu.my (A.A.S.G.); 6 Department of Mechanical Engineering, University Teknologi PETRONAS, Bandar Seri Iskandar 32610, Malaysia; haetham_19000233@utp.edu.my

**Keywords:** adsorption, heavy metals, magnetic biochar, iron oxides, kenaf fiber, synthesis, aqueous solution, cadmium

## Abstract

Development of strategies for removing heavy metals from aquatic environments is in high demand. Cadmium is one of the most dangerous metals in the environment, even under extremely low quantities. In this study, kenaf and magnetic biochar composite were prepared for the adsorption of Cd^2+^. The synthesized biochar was characterized using (a vibrating-sample magnetometer VSM), Scanning electron microscopy (SEM), X-ray powder diffraction (XRD), Fourier-transform infrared spectroscopy (FTIR), and X-ray photoelectron spectroscopy (XPS). The adsorption batch study was carried out to investigate the influence of pH, kinetics, isotherm, and thermodynamics on Cd^2+^ adsorption. The characterization results demonstrated that the biochar contained iron particles that help in improving the textural properties (i.e., surface area and pore volume), increasing the number of oxygen-containing groups, and forming inner-sphere complexes with oxygen-containing groups. The adsorption study results show that optimum adsorption was achieved under pH 5–6. An increase in initial ion concentration and solution temperature resulted in increased adsorption capacity. Surface modification of biochar using iron oxide for imposing magnetic property allowed for easy separation by external magnet and regeneration. The magnetic biochar composite also showed a higher affinity to Cd^2+^ than the pristine biochar. The adsorption data fit well with the pseudo-second-order and the Langmuir isotherm, with the maximum adsorption capacity of 47.90 mg/g.

## 1. Introduction

Water resources have been harmed by a variety of toxins, including heavy metals, dyes, surfactants, phenols, and other personal care chemicals [1,2,3]. Since heavy metal waste almost does not dissolve into harmless materials, it accumulates and is toxic to humans. It is also currently amongst the most important environmental concerns. Cadmium is one of the most harmful heavy metals, having been identified as a human carcinogen and teratogen with effects on the lungs, liver, and kidney [4,5,6].

Cadmium is naturally found in the environment as a result of the gradual erosion and abrasion of rocks and soils, as well as one-time events such as forest fires and volcanic eruptions [7,8,9]. As a result, it can be found naturally in the air, water, and soils. Cadmium is also unnaturally extracted from paint pigment, paints, garment manufacturing, battery manufacturing, gasoline manufacturing, and fertilizer manufacturing industries [10,11]. The permissible concentration of cadmium in drinking water has been set very low by the United States Environmental Protection Agency (the US EPA) at 0.005 mg/L, and even lower, at 0.003 mg/L, by the World Health Organization. Therefore, cadmium must be removed from wastewater before it can be discharged into environmental sources and contaminate the water resources [12,13,14].

Membrane-based separation, electrochemical deposition, chemical precipitation, coagulation, solvent extraction, ion exchange, and adsorption have all been explored to reduce the concentration of cadmium to an acceptable level and meet the environmental requirement [15]. Although all the aforementioned technologies are highly effective in removing heavy metals, they have significant drawbacks such as byproduct formation, large sludge production, and high energy requirements [16]. Adsorption is considered an attractive method due to its simplicity, cost-effectiveness, and the possibility of using it on a large scale [17,18,19]. Many adsorbents’ materials have been applied in industries, such as activated carbon, activated alumina, silica gel, molecular sieve carbon, molecular sieve zeolites, and polymeric adsorbents [3,20,21].

An ideal adsorbent material should have a large surface area, maximum adsorption power, mechanical stability, and the ability to be easily separated and regenerated [22]. Biochar has been considered as the promising adsorbent material due to its excellent adsorption capacities for heavy metals and organic pollutants in an aqueous solution [23,24]. Biochar is a solid product of biomass pyrolysis where pyrolysis occurs in the absence of oxygen at the high-temperature heating process for biofuel production [25,26]. Essentially, biochar production aims to produce energy or reduce the amount of biomass feedstock used. However, there has been a lot of emphasis on improving the pyrolysis conditions to increase yield and biochar properties [27,28,29].

The spent biochar suspended in an aqueous medium usually requires centrifugation and filtration measures for recovery. Such recovery process limits the use of biochar in wastewater treatment on a large scale. Furthermore, during these measures, pollutants adsorbed on the biochar may desorb, resulting in secondary pollution [30,31,32]. As a result, it is critical to address biochar’s flaws to increase its effectiveness in water contamination mitigation. Studies from the literature have proven that biochar can remove toxic compounds, but pristine biochar has a limited ability to adsorb heavy metals from wastewater [33]. Several types of feedstocks have been used and treated as biochar-based adsorbents for cadmium removal, such as alamo switchgrass [34], pine wood residues [35], pig manure [36], rice husk [29,37], dairy manure, oak wood [38], pine bark [38], and corn stalk [39].

Surface modification is a proper method to improve biochar properties. Biochar is typically rich in functional groups such as hydroxyl, carboxyl, carbonyl, and methylene on the surfaces of the pore system [40]. Biochar has a large surface area and strong adsorption efficiency that makes it attractive for the removal of heavy metals from wastewater [41]. Surface modification is categorized into activation and formation of composite [42]. Activation is usually carried out by physical or chemical activation, both of which are conventional and have been known about for a long time [43,44]. The biochar-based composite as part of biochar surface modification is usually carried out by modifying the biochar with other materials such as clay, carbonaceous materials, microorganism, organic compound, and metal oxide [45]. Those composites adjust the properties of biochar and improve their functional groups. Biochar surface modification technology is better than primary chemical and physical activation, where the surface modification creates new functional groups that are not present in either biochar or raw materials [42,46].

Biochar-based composite is still in its infancy. It is mainly used in industries, especially those targeting to adsorb specific pollutants where the biochar is specially synthesized to have a specific affinity to the limited pollutants [47]. Biochar has a negative surface charge with high surface area and large pore volume [48]. Those features allow biochar to be a sufficient and promising adsorbent due to distinct adsorption on oxygenated functional groups, electrostatic attraction to aromatic groups, and precipitation on the mineral of biochar [49,50,51]. The biochar-based metal oxide can extract negatively charged oxyanion from an aqueous solution by using the high surface area of biochar as a medium to embed metal oxide with contacting chemical properties [52,53,54].

Recently, biochar surface modification using metal oxide has been employed. Agrafioti et al. [55] used this technology and found out that soaking raw materials (rice husk) and biochar derived from municipal waste with iron powder FeCl_3_ before pyrolysis created a positive charge which helped to adsorb arsenic from aqueous solution. Vladimir et al. [56] used corncob biochar composite with FeCl_3_ and found a similar result to Agrafioti, in which the product efficiently adsorbed arsenic. Attempts have been made to use iron’s magnetic properties to produce magnetic biochar that can remove contaminants in pinewood biomass by compositing biochar with hematite, as reported by Wang et al. [57], which resulted in the double performance of sorption capacity in comparison with the pristine biochar. Akila et al. [58] and Khan et al. [59] used magnetic biochar to adsorb lead and cadmium from an aqueous solution and discovered that it was effective and easily separated.

In this study, magnetic biochar has been synthesized and developed from kenaf biochar and Fe_3_O_4_. The pristine and magnetic biochar were characterized by a vibrating-sample magnetometer (VSM), scanning electron microscope (SEM) X-ray diffraction (XRD), Fourier transform infrared spectroscopy (FTIR), and X-ray photoelectron spectroscopy (XPS). Adsorption isotherm, kinetics, and thermodynamic of Cd^2+^ with this magnetic biochar-based adsorbent have been investigated. Consecutively the effect of pH, initial concentration, and time on the adsorption process was also studied.

## 2. Materials and Methods

### 2.1. Materials

The kenaf fiber was collected from a kenaf plantation in Pahang, Malaysia. After collection, it was washed, sieved, and prepared for biochar production. Other chemicals, such as sodium hydroxide, sodium nitrate, hydrochloric acid, cadmium nitrate trihydrate, ferrous and ferric chloride, and ethanol, were purchased from (Sigma-Aldrich (M) Sdn Bhd, Selangor D.E, Malaysia). The chemicals were used as received without any treatment.

### 2.2. Methods

**Figure 1:**
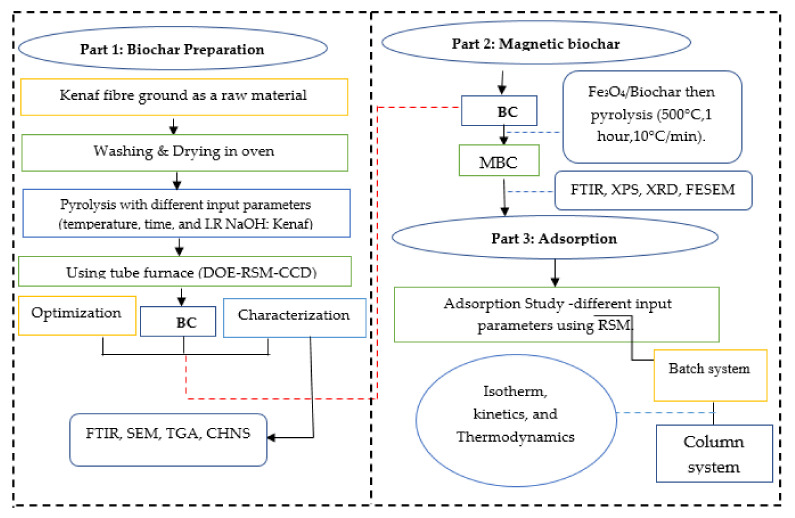
Summary chart of the composite magnetic biochar-based adsorbent preparation and testing. BC: biochar; MBC: magnetic biochar.

The preparation procedure of the composite magnetic biochar-based adsorbent was divided into two stages: biochar production and modification, followed by adsorption testing (Figure 1). In biochar production, the raw kenaf was first pyrolyzed with different input parameters such as pyrolysis temperature, heating time, and impregnation ratio (raw kenaf/NaOH). The resulting biochars were then modified with magnet materials to produce composite magnetic biochar. The biochar and magnetic biochar were characterized then applied further for the Cd^2+^ adsorption process.

#### 2.2.1. Biochar Preparation

**Figure 2:**
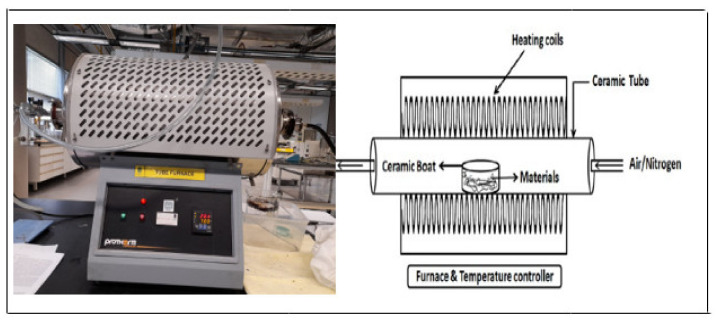
Schematic diagram of the tube furnace.

The raw kenaf fiber was washed continuously with distilled water to remove the impurities and dust from its surface, followed by drying in an oven at 105 ℃ for 24 h. The dried sample was crushed to the desired mesh size of 0.5 mm by passing through the respective sieves (Vibratory Sieve Shaker AS 200 Digit cA Model). It was then stored in an airtight container at room temperature before use. Kenaf biochar was prepared by slow pyrolysis. The preparation parameters were preoptimized at pyrolysis temperature of 550 ℃, 180 min reaction time, and 1:1 impregnation ratio with sodium hydroxide using tube furnace shown in in Figure 2 [60].

#### 2.2.2. Magnetic Biochar Preparation

**Figure 3:**
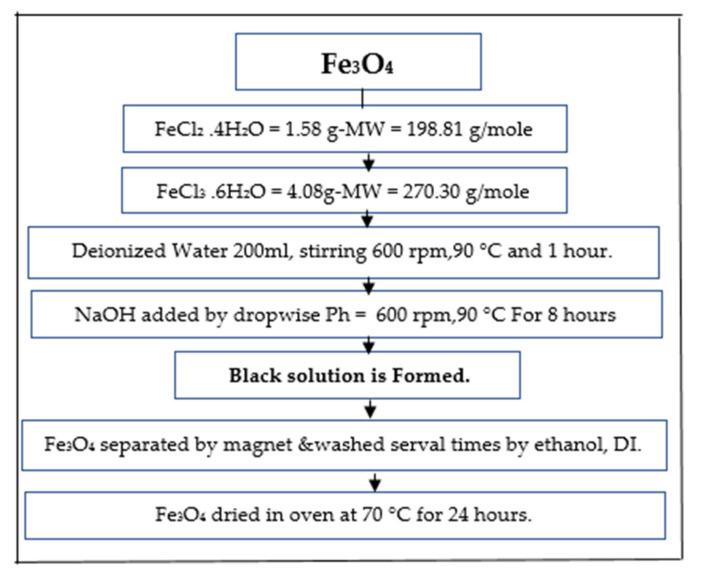
The synthesis procedure of Fe_3_O_4_ nanoparticles.

Magnetic biochar was synthesized by incorporating Fe_3_O_4_ nanoparticles. It was precipitated using methods employing ferric chloride (FeCl_3_.6H_2_O) and ferrous chloride (FeCl_2_.4H_2_O) with a ratio of 2:1, according to a protocol summarized in Figure 3 [61,62]. Subsequently, the prepared Fe_3_O_4_ was mixed with kenaf biochar and added into 150 mL deionized water before being agitated for 45 min. This composite was sonicated and evaporated to dryness in a water bath for three hours at a constant temperature (100 ℃).

The composite magnetic biochar was subjected again to calcination at 500 ℃ in N_2_ flow (600 cm^3^/min) for 1 h at a 10 ℃ /min heating rate. After being cooled down to room temperature, the composite magnetic biochar was then washed again with distilled water to neutralize the pH.

### 2.3. Characterizations

The moisture content, volatile matter, ash content, and fixed carbon content were determined proximally using ASTM D7582-10 techniques. The contents of carbon (C), nitrogen (N), hydrogen (H), sulfur (S), oxygen (O), H/C, and O/C were detected using the Vario Micro Element Analyzer. The kenaf fiber biochar samples were cleaned and dried before being analyzed. The biochar samples were placed in the oven at 105 ℃ for 3 h for the measurement of moisture content. The volatile matter was determined by placing a closed crucible containing 2 g of biochar samples in a carbonite furnace at 950 ℃ for 10 min. The ash content was determined by placing a crucible containing 2 g of biochar sample in the furnace at 850 ℃ for 1 h. All these amounts were assessed using the difference between the initial and final weights.

The functional group analysis of the biochar samples was determined using Fourier-transform infrared spectroscopy (FTIR; Operant LLC, Madison, WI, USA). The FTIR spectra were recorded in wavelengths ranging from 400 to 4000 cm^−1^. The FTIR plots were investigated to comprehend the possibility of a functional group’s change. Scanning electron microscopy (SEM) coupled with energy-dispersive X-ray spectroscopy (EDS) was used to visualize the microstructure of biochar-based adsorbent and the elemental surface composition of the biochar-based adsorbent samples. SEM images were accomplished to detect the morphology and structural changes of the adsorbents. X-ray photoelectron spectroscopy (XPS) was employed to determine the mechanism of adsorption of samples with XPS spectrometers (Thermo Fisher Scientific, Waltham, MA). The specific surface area and the pore size of biochar were assessed using a Micromeritics ASAP 2020 analyzer. A vibrating-sample magnetometer was used to analyze the magnetic properties of the magnetic biochar and raw synthesized iron oxide.

The pH values of the biochar adsorbents at the point-of-zero charges (PZC; pH_pzc_) were obtained using the solid addition method (Balistrieri and Murray, 1981) [63]. By adding 1M NaOH or 1M HCl to the starting pH value (pH_i_) of 0.1 M NaCl, the pH was raised from 2 to 11; 50 mL of this solution was then applied to a series of flasks, and 0.1 g biochar was added to each flask. The flasks were immediately capped, and the suspensions were agitated at 200 rpm for 3 h before the supernatant’s final pH value (pH_f_) was measured. The difference between pH_i_ and pH_f_ (Ph = pH_i_ pH_f_) was plotted against pHi, and the point where the curve intersected with pH_i_ was pH_PZC_.

### 2.4. Adsorption Study

A 1000 mg/L Cd (II) stock solution was prepared by dissolving 2.744 g of cadmium nitrate tetrahydrate in deionized water. Then, the stock solution was diluted to different concentrations for batch studies. The solution was treated with different parameters to obtain the best adsorption performance. The batch adsorption studies were conducted using an Erlenmeyer flask by loading the biochar in a cadmium aqueous solution. The fixed parameters were the dose of the adsorbent, the pH of the adsorbent, and the stirring speed. Parameters such as contact time, solution pH, initial concentration, and temperature were varied.

The effect of pH on the adsorption was identified by adjusting the pH value from 2 to 8 by adding 0.1 M hydrochloric acid and 0.1 M sodium hydroxide to change it from acidic to alkaline conditions. The sample was filtered using syringe filters and Whatman filter paper and analyzed for absorbance using atomic absorption spectrometry (AAS, Hitachi Z-5000). The removal efficiency (η, %) and adsorption capacity of magnetic biochar were determined using Equations (1) and (2):

(1) $$\eta =\frac{\left(C_{i\;}-\;C_{f}\right)}{C_{i}}\times 100\%$$

(2) $$q_{e}=\frac{\left(C_{i\;}-\;C_{f}\right)V}{m}$$

where $C_{i}$ and $C_{f}$ are Cd^2+^ concentrations (mg/L) at the initial and final stage, $q_{e}$ is the adsorption capacity in mg/g, m denotes the quantity of magnetic biochar (g), and V is the volume of cadmium solution (L).

### 2.5. Kinetics, Isotherm, and Thermodynamic Analysis

Three different temperatures of 25, 35, and 45 °C were selected for evaluating the thermodynamics of adsorption. Table 1 specifies linearized forms of several isotherms of the Temkin, the Freundlich, and the Langmuir models. For adsorption kinetic analysis, various initial concentrations (of 5, 10, 20, 40, 60, 80, and 100 mg L^−1^) were selected for the pseudo-first-order and pseudo-second-order kinetic models, as provided in Table 1. The pseudo-first-order (PFO) and pseudo-second-order (PSO) kinetic models are the most common models used to understand the adsorption mechanism and potential rate-limiting steps. The two models’ formulas are listed in Table 1.

The Temkin, the Freundlich, and the Langmuir constants are denoted by K_T_, K_F,_ and K_L_, with corresponding units of L/mg, mg/g, and L/mg. Parameter B denotes the Temkin constant (J/mol).

## 3. Results and Discussion

### 3.1. Biochar Characteristic

#### 3.1.1. Ultimate and Physical Properties

Table 2 summarizes the chemical and physical properties of the prepared biochars. The carbon, hydrogen, nitrogen, ash, and oxygen content of raw kenaf were 39.2, 5.12, 0.35, 16.32, and 43.6%; biochar were 67.52, 1.27, 1.23, 10.80, and 19.18%; magnetic biochars were 58.32, 1.12, 1.06, 13.75, and 25.75%, respectively.

The iron content of magnetic biochar was 8.34%, indicating that the surface of magnetic biochar was highly loaded with iron. The carbon content rose from 39.2 to 67.52%, while the oxygen content declined from 43.6 to 19.177%, indicating that the experimental conditions of the pyrolysis temperature and activation steps were the only significant contributors to the improvement of the biochar properties. The O/C ratio indicates the polarity and abundance of the polar oxygen-containing surface functional groups in biochar. A higher O/C ratio indicates more polar functional groups, which actively take part in the adsorption of cadmium. On the other hand, the H/C ratio indicates an aromaticity and stability of the biochar [69]. The magnetic biochar had a lower carbon content of 58.32% and high iron content compared to the pristine biochar, indicating that the carbon was significantly blended with the iron.

The specific surface area of raw kenaf, biochar, and magnetic biochar were 4.50, 117.70, and 175.55 m^2^ g^−1^, respectively. These results indicate that the impregnation of iron oxide with biochar influenced the pore opening activation and pore structure of the pristine biochar, as also reported earlier [60].

#### 3.1.2. Magnetic Properties

**Figure 4:**
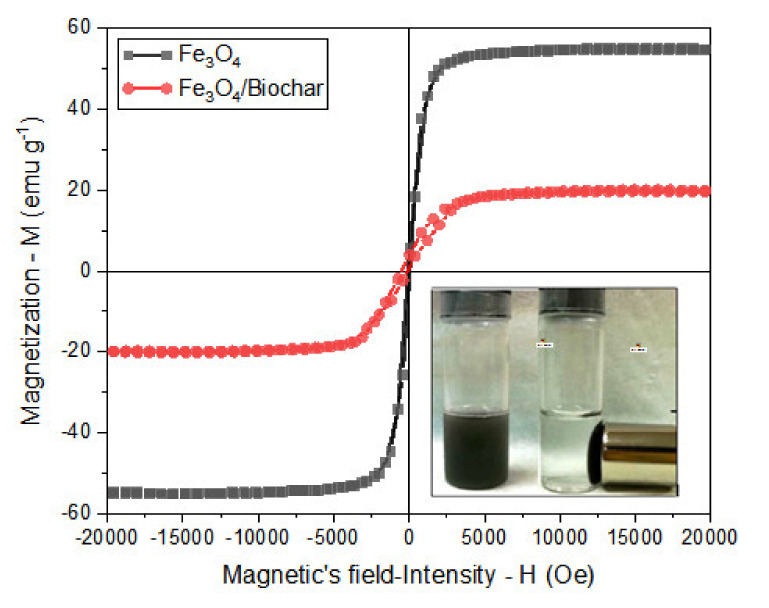
Magnetization curves of synthesized Fe_3_O_4_ nanoparticles and the magnetic biochar (Fe_3_O_4_/biochar). The inset shows the Fe_3_O_4_ nanoparticle under influence of a magnetic field.

The magnetism curve in Figure 4 indicates that the magnetic biochar had superparamagnetic properties with a magnetism M value of 19.98 emu g^−1^ and a retentivity (Mr) value of 1.62 emu g^−1^. When comparing the magnetic properties of iron oxide and magnetic biochar, the magnetism and retentivity values of Fe_3_O_4_ and magnetic biochar were 55.10 emu/g and 3.5438 emu g^−1^, while the magnetism and retentivity values of the magnetic biochar were 19.98 emu g^−1^ and 1.62 emu g^−1^. These findings prove that after incorporating magnetic particles, the magnetic biochar gained sufficient magnetism for recovery after use.

#### 3.1.3. Microstructure Analysis

**Figure 5:**
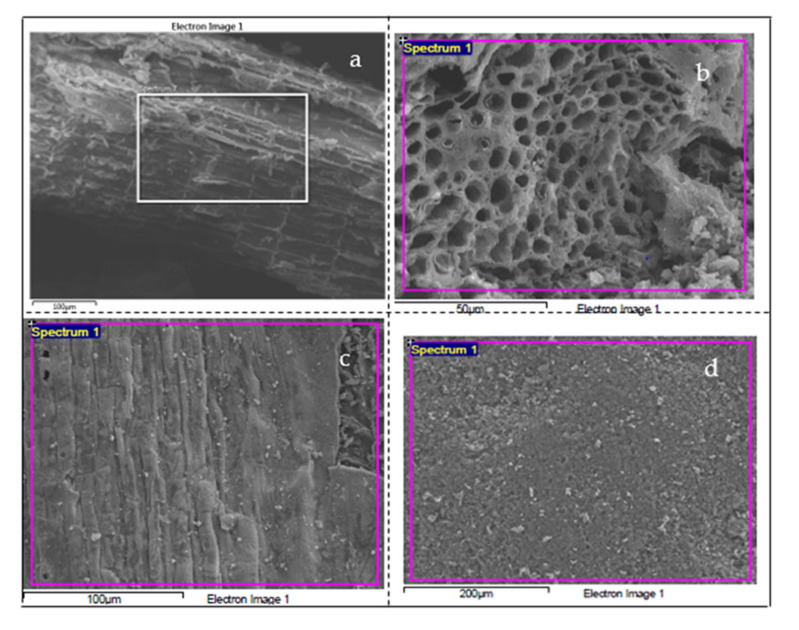
Scanning electron microscopy (SEM) images of (**a**) raw kenaf, (**b**) biochar, (**c**) magnetic biochar, and (**d**) the spent magnetic biochar after adsorption test.

Figure 5 illustrates the SEM images of raw kenaf, the biochar, and the magnetic biochar, as well as the magnetic biochar after adsorption. When compared to raw kenaf, the morphology of the biochar changed. The biochar SEM image in Figure 5b reveals micropores, macropores, and mesopores in several shapes. The biochar had a honeycomb-like morphology, with cylindrical holes intertwined with several big holes [70]. Many pore mouths in ordered manner can be seen over the surface of the biochar. The biochar was then subjected to magnetism and took on new shapes, indicating that surface modulation improved the textural properties and made it possible for Fe to adsorb onto the surface. The magnetic biochar SEM image (Figure 5a,d) shows particles trapped on the surface with plate-like roughness and distorted morphology containing sharp edges and corners, as seen in Figure 5c,d, as opposed to pristine biochar and raw kenaf, because the iron particles were filled in within the pristine biochar matrices which indicated good mechanical bounding [71]. 

#### 3.1.4. XRD Analysis

**Figure 6:**
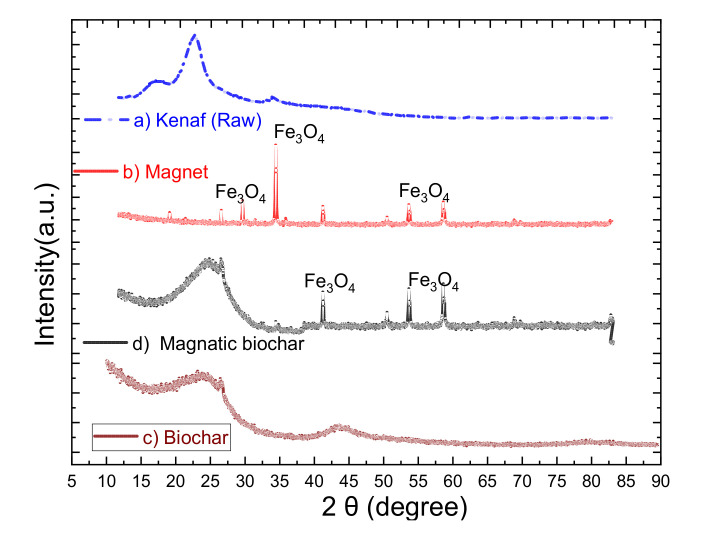
XRD patterns of (**a**) kenaf (raw), (**b**) Fe_3_O_4_, (**c**) biochar, and (**d**) magnetic biochar.

Figure 6 shows the XRD analysis performed over the range of 2θ = 10°–90° to classify the crystallographic structure changes of biochar, magnetic biochar, and the raw materials (kenaf and magnet). The XRD data (Figure 6d) shows that the magnetic biochar was amorphous, so the magnetite ores were loaded on the surfaces of the biochar. Amorphous biochar, which probably had a relatively homogeneous structure, could prevent the aggregation of the magnet nanoparticles and, therefore, maximize the adsorption capacity. According to Kang et al. [72], a good adsorption capacity is achieved by using amorphous adsorbent. Due to the thermochemical reactions between iron and carbon during pyrolysis, the amorphous carbon peak in the magnetic biochar pattern weakened greatly, resulting in a macrocrystalline graphite crystal lattice flaw. The magnetic biochar pattern’s characteristics peaks at 42.5°, 50.4°, 53.7°, and 57.8° show the presence of Fe in the biochar. The Fe_3_O_4_ phase was strongly crystalline and deposited on the composite’s surface, as shown by the sharpness of the XRD peaks, and these findings are in agreement with studies done by Hongwei et al. [73] and Tassya et al. [74].

#### 3.1.5. Surface Functional Group

**Figure 7:**
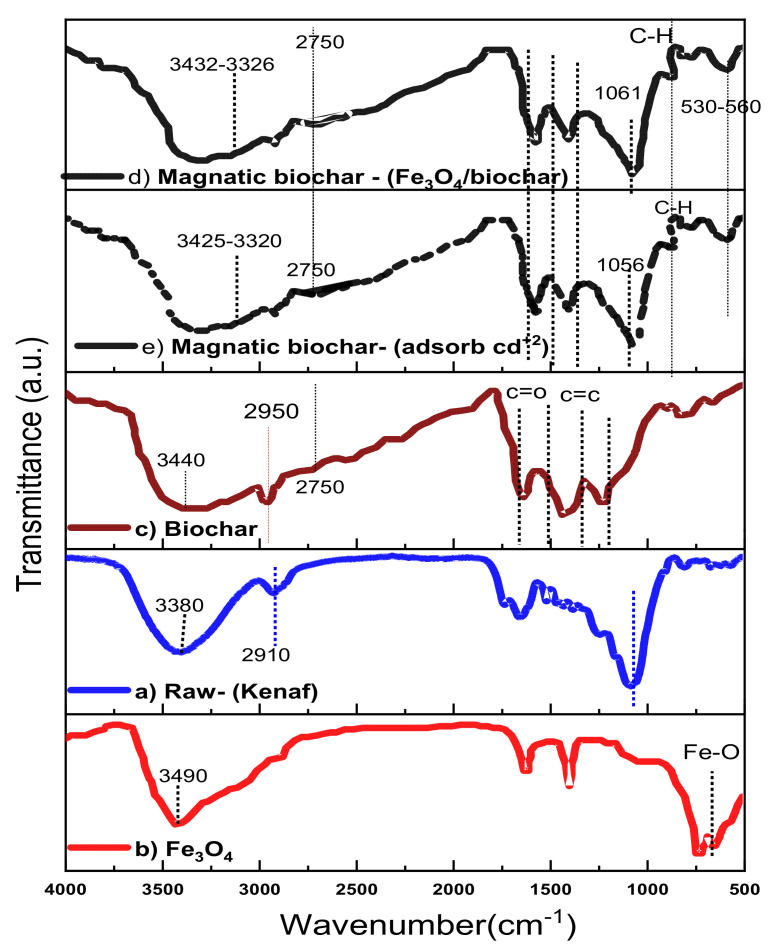
FTIR spectra of (**a**) kenaf (raw), (**b**) Fe_3_O_4_, (**c**) biochar, (**d**) magnetic biochar, and (**e**) magnetic biochar (after Cd(II) adsorption).

Figure 7 shows the FTIR spectra indicating that adsorption of metals on the surface of biochar is influenced by the pyrolysis temperature, the raw materials used, and the activation agent, as also reported elsewhere [75]. Figure 7c shows that there was an improvement due to pyrolysis temperature indicated by a peak at 1483 cm^−1^ corresponding to the C=O bonds and aromatic hydrocarbon. The broad and strong peak at 3440 cm^−1^ indicates the O–H bond. Meanwhile, the peak at 1375 cm^−1^ represents the C=C bond. The peak at 675 cm^−1^ represents the C–H bending.

Magnetic biochar in Figure 7d,f show the stretching vibration of hydroxyl (O–H) at 3320 cm^−1^–3425 cm^−1^ and 3325 cm^−1^–3432 cm^−1^, respectively [76]. Similarly, the magnetic biochars have stretching vibration of lactone, carbonyl, ester, carboxylic, and aromatic structure (C=O) at 1615 cm^−1^–1580 cm^−1^. The peak at 3320 cm^−1^–3425 cm^−1^ for the magnetic biochar significantly weakened after adsorption, where there was a significant shift from 3320 cm^−1^–3425cm^−1^ to 3325 cm-^1^–3432 cm^−1^ and that shift occurred due to the interaction between Cd(II) and hydroxyl groups which formed on the magnetic biochar [77]. The magnetic biochar has the highest number of pores and high peaks ranging from 530 cm^−1^ to 560 cm^−1^, indicating the presence of Fe particles on its surface. Furthermore, the broad stretching vibration peaks ranged from 2750 cm^−1^–2950 cm^−1^ could be the C–H groups of nonionic carboxylic groups, while peaks at 1650 cm^−1^–1700 cm^−1^ indicated the presence of C=O groups as a result of the pyrolysis process [78,79].

#### 3.1.6. XPS Analysis

**Figure 8:**
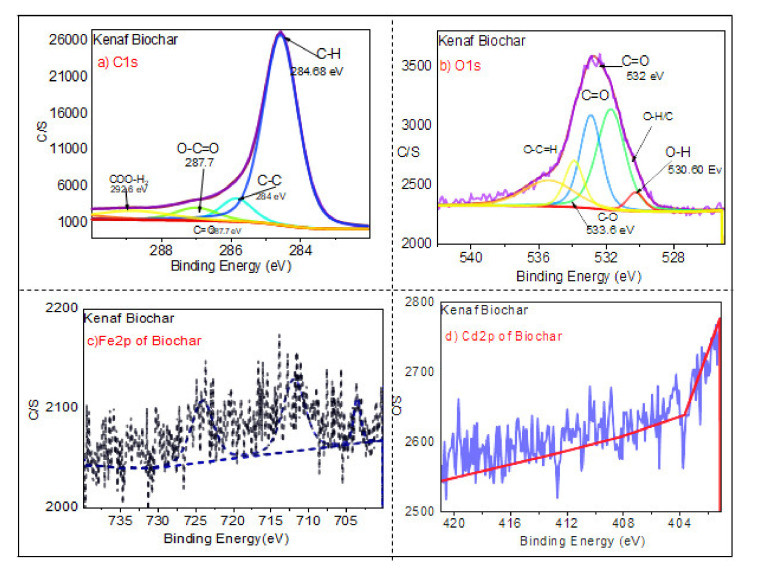
XPS analysis of the biochar by showing (**a**) C1s spectrum, (**b**) O1s spectrum, (**c**) Fe2p spectrum, and (**d**) Cdp2 spectrum.

**Figure 9:**
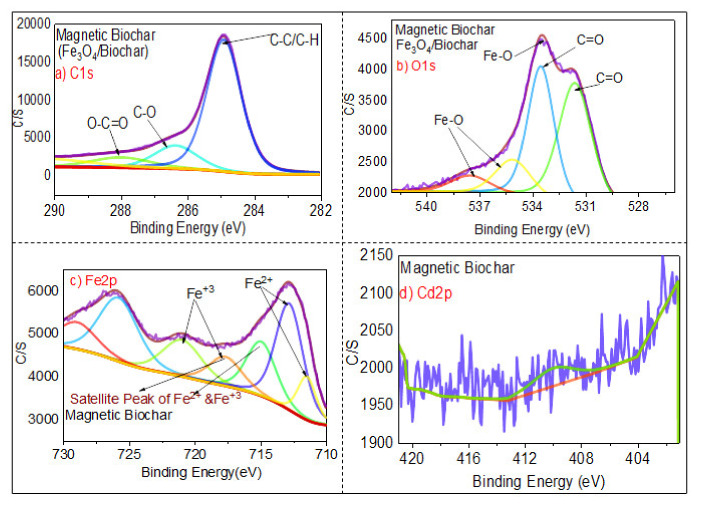
XPS analysis of the magnetic biochar by showing (**a**) C1s spectrum, (**b**) O1s spectrum, (**c**) Fe2p spectrum, and (**d**) Cdp2 spectrum.

**Figure 10:**
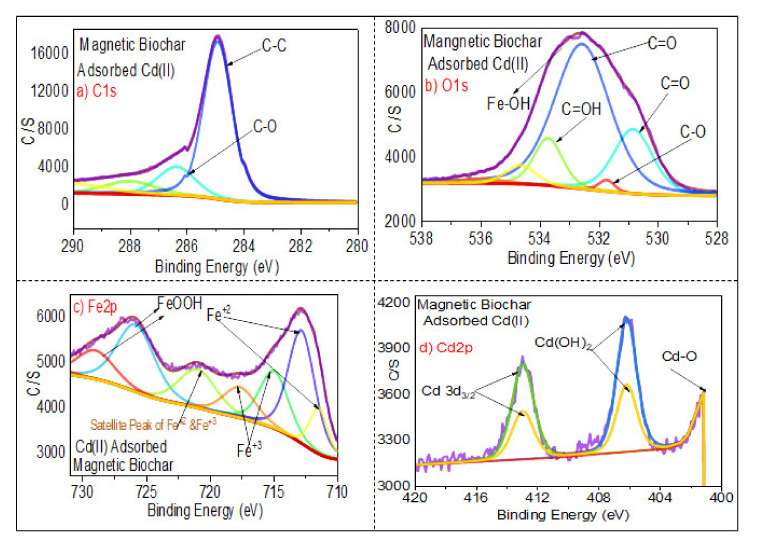
XPS analysis of the spent magnetic biochar by showing (**a**) C1s spectrum, (**b**) O1s spectrum, (**c**) Fe2p spectrum, and (**d**) Cdp2 spectrum.

Figure 8, Figure 9 and Figure 10 show the XPS spectra of the biochar, the magnetic biochar, and the spent magnetic biochar, respectively. The figures show the XPS high-resolution spectra of C1s, O1s, Fe2p, and Cdp2 spectrum regions on the adsorbents. Figure 8a,b and Figure 9a,b show the region of C1s spectrum and O1s spectrum indicating that the presence of carbon with little oxygen in the pristine biochar increases the oxygen and presence of Fe after impregnation with Fe_3_O_4_. Figure 8a shows that there was a significant difference with carbon-containing group peaks observed for the carbon peaks of the biochar. As shown at 284 eV in Figure 8a, carbon was bound to carbon and hydrogen; at 284.68 eV, carbon was bound to oxygen or nitrogen (carbonyl, amines, alcohols, or amides); at 287.7 eV, carbon formed two single bonds or one double bond with oxygen (acetals, amides, carboxylates, hemiacetals, and acetals); at 292.6 eV, carbon formed one double bond and one single bond with oxygen (carboxyl group). Similarly, in Figure 8b, the O1s peaks revealed oxygen-containing groups, with the existence of O=C, C–O, C=O, and O–H confirmed by the XPS spectrum of O1s at 536, 533.6, 531.2, and 530.60 eV, respectively.

Figure 9 shows the XPS analysis of magnetic biochar spectra C1s spectrum, O1s spectra, Fe2p spectrum, and Cdp2 spectrum. The C1s spectrum and O1s spectra indicate that the carbon percentage decreased and oxygen percentage increased after the impregnation of biochar with iron. Figure 9c displays XPS spectra with high resolution, revealing the existence of Fe2p_1/2_ at 715.5–725 eV and Fe 2p_2/3_ at 711–714.5 eV. These findings demonstrate the presence of Fe^3+^ and Fe^2+^, which appeared as a result of mixing biochar with iron oxide. Satellite peaks of Fe^3+^ and Fe^2+^ were found at the range of binding energy of 715–720 eV, which is similar to the results of Naeemeh et al. [80] and Haixia Wu et al. [81]. Figure 10c shows that after adsorption of Cd(II), there was a significant displacement of Fe 2p binding energy, implying the occurrence of coordination reactions between Fe and Cd.

Figure 10 shows the broad peaks of magnetic biochar (after adsorption), C1s spectrum, O1s spectrum, Fe2p spectrum, and Cdp2 spectrum. In comparison to the O1s spectrum of the pristine biochar (Figure 8b) showing the presence of oxygen in the form of organic oxygen (C–OH)/C–O–C) and inorganic oxygen (Fe–O/Cd–O), Figure 10b shows strong peaks in the O1s region, indicating several chemical states of oxygen such as organic oxygen (C–OH)/C–O–C) and inorganic oxygen (Fe–O/Cd–(C–O–C). Meanwhile, Figure 10d shows that the Cd 3d3/2 core-level spectrum has two peak components with binding energies of about 413.5 eV, 406 eV, and 402 eV, which can be attributed to Cd–O and Cd (OH)_2_ species, respectively. The findings show that Cd(II) deposition and chelation played a significant role in the adsorption mechanism [82].

### 3.2. Adsorption Kinetic Analysis

**Figure 11:**
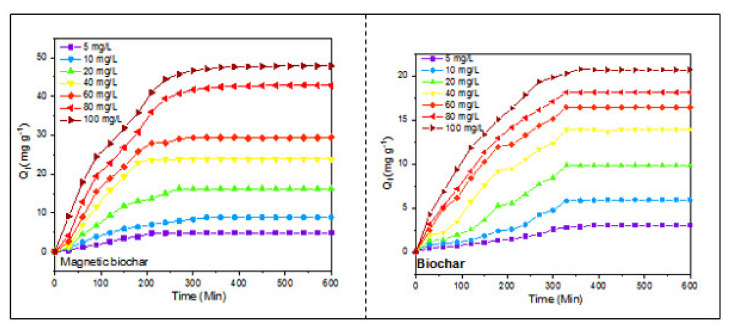
Adsorption kinetics of Cd^2+^ with biochar and magnetic biochar.

The kinetics study was applied to Cd^2+^ adsorption using the biochar and the magnetic biochar. Figure 11 shows the evolution of adsorption as function of time under initial Cd^2+^ concentrations (of 5, 10, 20, 40, 60, 80, and 100 mg L^−1^). The Cd^2+^ adsorption onto the biochar and the magnetic biochar rapidly improve in the first two hours but at different rates. The adsorption on the magnetic biochar in the first two hours reaches double the adsorption on the biochar. Overall understanding from Figure 11 indicated that most of the Cd^2+^ ions could be adsorbed within 4 h until reaching the equilibrium when the internal active adsorption sites were fully occupied. The magnetic biochar adsorbed Cd^2+^ more than the biochar, even in the same period, suggesting that it had better adsorption properties than the biochar. The surface area and pore volume of magnetic biochar was greater than the surface area and pore volume of biochar of 175.55 m^2^ g^−1^, 0.102366 cm^3^ g^−1^, and 117.70 m^2^ g^−1^, 0.063884 cm^3^ g^−1^, respectively.

The adsorption kinetic characteristics for biochar and magnetic biochar onto cadmium adsorption are shown in Figure S1 and Table S1 (Supplementary Materials). The pseudo-first-order model has a lower correlation coefficient than the pseudo-second-order model, indicating that the latter fits better to explain the adsorption kinetic which associates with the chemisorption mechanism. Furthermore, the calculated adsorption capacity values with the pseudo-second-order model were close to ones obtained from the experiments, which confirmed the good fittings of the pseudo-second-order model.

Table S1 shows that the pseudo-second-order model is more suitable for the rate-determining step than the pseudo-first-order model, which is comparable to the study by Chowdhury et al. [83], and includes valence forces from the exchange or sharing of electrons between the adsorbate and adsorbent. The estimated value of Q_cal_ using the pseudo-second-order model was close to the experimental value, confirming the pseudo-second-order model’s ability to fit the kinetic adsorption data. The adsorption capacity constant (K_2_) of magnetic biochar was higher than the adsorption capacity constant of pristine biochar (K_2_), indicating that fast sorption of Cd^2+^ to magnetic biochar where K_2_ values at initial concentration 100 ppm for magnetic biochar and pristine biochar were 0.127 (g/mg.min) and 0.025 (g/mg.min), respectively.

### 3.3. Adsorption Isotherm Analysis

The adsorption isotherms of treatment of Cd^2+^-containing solutions were successfully determined using experimental adsorption results by linearly fitting models of Temkin, Freundlich, and Langmuir. The analysis of adsorption isotherms is very important for understanding the mechanism of the adsorption process and the principle and mathematical derivation of adsorption characteristics. The result of isotherm analysis is shown in Figure S2 and Table S2 (Supplementary file). The maximum R^2^ values of the tested isotherms indicated that the model mostly suits the experimental results [2,84]. Comparing the correlation coefficients for the three isotherm models obtained for biochar and magnetic biochar, the most suitable is the Langmuir model. It shows that the magnetic biochar had superior adsorption ability than the biochar due to its higher surface area, stronger affinity toward Cd^2+^, and a larger number of actives sites. The K_L_ of the magnetic biochar of 0.09798 L mg^−1^ is higher than the K_L_ of the biochar of 0.035196 L mg^−1^. It confirms the stronger affinity of magnetic biochar to the Cd^2+^ [85]. The high adsorption capacity of magnetic biochar might be due to the good affinity of cadmium to iron oxide on the magnetic biochar, which was also presented by XPS characterization, which generates a stable inner sphere to the surface functional groups(–COOH, –OH) [86]. In Table S2, it is proved that the Langmuir isotherm model fits well, with a correlation R^2^ value of 0.963 and 0.995 for both biochar and magnetic biochar, respectively, while the Freundlich and Temkin isotherm model correlation R^2^ values for biochar and magnetic biochar were 0.953, 0.983, 0.773, and 0.913, respectively.

### 3.4. Effect of pH

**Figure 12:**
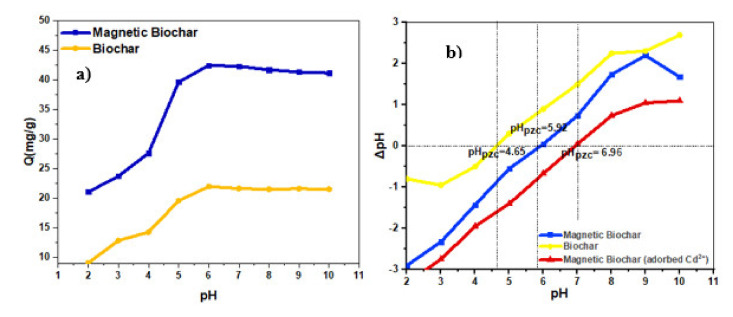
(**a**) Effect of solution pH on adsorption capacity of biochar and magnetic biochar and (**b**) point-of-zero charges of biochar, magnetic biochar, and magnetic biochar (adsorbed Cd^2+^).

pH is one of the significant parameters that influence the adsorption capacity. The adsorption capacity might be influenced by pH in different aspects: concentration of metal ions or electrical surface charge on the adsorption [87,88]. The range of pH was between 2.0–10 to study the influence of pH on adsorption capacity, while the other parameters were kept constant. Figure 12a shows the effect of the solution pH on the adsorption of Cd^2+^ on the magnetic biochar and the biochar. The trend indicated that the adsorption capacity for both adsorbents increases as pH increased to 6, then it slightly decreased. The adsorption capacity of magnetic biochar and biochar was 9.65 mg g^−1^ and 21.1 mg g^−1^, respectively, at pH 2.0, and later reached the maximum value of 43.92 mg g^−1^ and 22 mg g^−1^, respectively, at pH value 6.0. The high adsorption capacity of adsorbents on cadmium ions occurred around pH ranging from 5.5 to 6.0. This happened because the surface of biochar becomes saturated with hydrogen ions at lower pH levels, and this saturation prevents the removal of cadmium ions owing to the mutual repulsion between cadmium and hydrogen ions [89,90]. Above pH 6.0, cadmium precipitation usually occurs. This is because insoluble cadmium hydroxides such as Cd (OH)_2_ and Cd (OH)^3-^ begin to precipitate out of solution at higher pH values. This must be avoided to discriminate between sorption and precipitation [90].

The results of this study agree with the ones obtained by Usman et al. [91], which stated that the adsorption capacity increased with increased pH solution. This might be attributed to a lower level of competition between protons and Cd^2+^ for accessible sorption sites. Furthermore, as pH rises, the number of binding sites increases, hence increasing metal adsorption on the adsorbent surface. Figure 12b shows the pH_PZC_ result which indicated that the pH_pzc_ of magnetic biochar was 6.92. If pH_pzc_ > pH, the adsorption capacity might be reduced due to possible electrostatic repulsion between the positive change of magnetic biochar and the positive charge of cadmium ions. Figure 12b shows that after adsorption, the magnetic biochar changed toward the positive, indicating that the cadmium ions were efficiently adsorbed as inner-sphere surface complexes or cations that may neutralize the surface’s negative charge [59,92].

### 3.5. Effect of Temperature

Thermodynamic adsorption study is crucial because it determines whether the adsorption process is a spontaneous process or an intended process. The enthalpy (ΔH°), entropy (ΔS°), and change in Gibbs free energy (ΔG°) were calculated based on the following equations:

(3) $$\Delta G{}^{\circ}=-{\mathrm{RTInK}}_{L}$$

(4) ΔG°=ΔH°−TΔS°

Table 3 shows the thermodynamic parameters of Cd^2+^ adsorption using the magnetic biochar at different temperatures. It proves that the adsorption process was spontaneous and endothermic, with negative values of Gibbs free energy (ΔG°) and positive values of ΔH° and ΔS°. Such a mechanism is thermodynamically feasible and conforms to a chemical reaction in Cd^2+^ adsorption because the ΔG° is negative. Additionally, when the ΔG° reduced, the temperature climbed (−3.96, −4.42, and −4.70 kJ mol^−1^), showing that the feasibility rose with temperature. The temperature affects the Cd^2+^ adsorption of magnetic biochar, as demonstrated in Figure S3, which proves that ion mobility increases with increasing temperature.

### 3.6. Performance Assessment of Magnetic Biochar in Comparison with Literature

Table 4 shows the performance of magnetic biochar for Cd^2+^ adsorption compared to magnetic adsorbents reported in the literature. The adsorption capacity of magnetic biochars for Cd^2+^ ranges from 6.34 to 197.76 mg/g [93]. The adsorption capacity of Fe_3_O_4_/kenaf fiber biochar for Cd^2+^ adsorption is better than the magnetic oak wood biochar, magnetic coconut shell biochar, magnetic Douglas fir biochar, and other magnetic adsorbents. However, the adsorption capacity of chitosan-modified magnetic biochar composite is higher than that of Fe_3_O_4_/kenaf fiber biochar. The performance comparison results indicated and reconfirmed that magnetic biochar composites from the kenaf with Fe_3_O_4_ are positively capable of being an efficient adsorbent for cadmium removal from aqueous environments.

## 4. Conclusions

The magnetic biochar derived from kenaf fiber showed better performance than pristine biochar, with twice-higher adsorption capacity. The magnetic biochar demonstrated enormous physicochemical properties such as surface area, pore volume, more oxygen-containing functional groups, and fine pore structure. The magnetic biochar offers a high adsorption capacity of Cd^2+^. The adsorption process was favored at 5–6 pH values. The Langmuir model best suited the experimental data with the correlation coefficient R^2^ of >0.99, compared to other models. The R^2^ for the pseudo-second-order model was higher than the pseudo-first-order model for all tested initial Cd^2+^ concentrations, implying predominance of the chemical adsorption process. The overall results suggest that magnetic biochar is fully suited for Cd^2+^ removal. Further study can focus on the magnetic induced separation of the magnetic adsorbent using an external magnetic field.

## Figures and Tables

**Table 1:** Equilibrium and linearized equations of isotherm and kinetic models.

Model	Equilibrium	Linearized	Ref.
Langmuir	$Q_{e}=\frac{q_{m}bC_{e}}{1+bC_{e}}$	$\frac{C_{e}}{q_{e}}=\frac{1}{q_{m}b}+\frac{C_{e}}{q_{m}}$	[64]
Freundlich	$Q_{e}=K_{f}C_{e}^{n}$	$\log q_{e}=Log\;K_{F}+\frac{1}{n}\log C_{e}$	[65]
Temkin	$q_{e}=\frac{RT}{b}\ln\left(K_{T}C_{e}\right)$	$Q_{e}=B\;Ln\;K_{T}+B\;Ln\;Ce$	[66]
Pseudo-first-order	$q_{t}=q_{e}\left(1-e^{-k_{1}t}\right)$	$\ln\left(q_{e}-q_{t}\right)=lnq_{e}-k_{1}t$	[67]
Pseudo-second-order	$q_{t}=\frac{q_{e}^{2}k_{2}t}{\left(1+q_{e}k_{2}t\right)}$	$q_{t}=q_{e}\left(1-e^{-k_{1}t}\right)$	[68]

**Table 2:** Selected chemical and physical properties of biochar and magnetic biochar.

Adsorbent	C%	H%	N%	O^*^%	Fe%	ash	O/C	H/C	S_BET_ (m^2^ g^−1^)	Pore Volume (cm^3^ g^−1^)	Pore Width (nm)
Kenaf (Raw)	39.2	5.12	0.35	43.6	N/A	16.32	1.12	0.130	4.50	0.009819	2.07
Biochar (550 °C)	67.52	1.27	1.23	19.18	N/A	10.80	0.284	0.0188	117.70	0.063884	2.17098
Magnetic Biochar	58.32	1.12	1.06	25.75	8.34	13.75	0.442	0.019	175.55	0.102366	2.33242

* calculated by difference; S_BET_: surface read calculated by Brunauer–Emmett–Teller (BET) analysis.

**Table 3:** Thermodynamic parameters of Cd^2+^ adsorption on the magnetic biochar at different temperatures.

T(K)	Q_e_ (mg g^−1^)	InK_L_	ΔG° (KJ mole ^−1^)	ΔH° (kJ mole ^−1^)	ΔS° (KJ mole ^−1^)
298	44.60	1.60	−3.96	8.69	0.042
308	45.80	1.73	−4.42		
318	47.30	1.82	−4.70		

**Table 4:** Performance of magnetic biochar for Cd^2+^ adsorption compared to literature-based magnetic adsorbents.

Adsorbent	SSA (m^2^ g^−1^)	pH	Cd^2+^ Adsorption Capacity (mg g^−1^)	Ref.
Magnetic oak wood biochar	8.80	5	7.40	[94]
(iron oxide/tangerine peel biochar)	-	4	15.5	[95]
Magnetic coconut shell biochar	834	4.8	4.77	[96]
Magnetic spent coffee ground biochar	3.60	7	10.42	[97]
Magnetic hollow porous oval shape NiFe_2_O_4_	-	5	16.65	[98]
Grape husk/FeSO_4_.7H_2_O	127	5	38.3	[99]
Algal-magnetic biochar	63.33	5	19.40	[100]
Fe_3_O_4_@FePO_4_	-	7	13.51	[49]
Magnetic Douglas fir biochar	460	5	11	[58]
(Mangosteen peel/Fe_2_o_3_) biochar	-	7	45.662	[101]
Magnetic chicken manure biochar	5.44	6	8.1	[102]
Chitosan modified magnetic biochar composite	112.33	6	93.72	[103]
Corn straw waste @ferric nitrate	313.90	2–10	46.15	[59]
Fly ash/Fe_3_O_4_	-	5	9.65	[104]
Fe_3_O_4_/kenaf fiber biochar	175.55	6	47.90	This study

## Data Availability

The data presented in this study are available from the corresponding author upon reasonable request.

## Supplementary Materials

The following are available online at https://www.mdpi.com/article/10.3390/ijerph18157949/s1, Figure S1: Adsorption kinetic parameters for the biochar and magnetic biochar –Cd^2+^ adsorption system, Table S1: Isotherm parameter of Cd^2+^ adsorption with biochar and magnetic biochar, Figure S1: Kinetic models of the (a) pseudo-first order and (b) pseudo-second order, Figure S2: Fitting of isotherm models: (a) Langmuir isotherm, (b) Freundlich isotherm, and (c) Temkin isotherm, Figure S3: Influence of temperature on Cd^2+^ adsorption by magnetic biochar.
