# Supplementary material for: Pristine and Magnetic Kenaf Fiber Biochar for Cd2+ Adsorption from Aqueous Solution
Source: Int J Environ Res Public Health. 2021 Jul 27;18(15):7949. doi: 10.3390/ijerph18157949 (PMC8345446; doi:10.3390/ijerph18157949)
Supplement: Supplementary file 1 [file ijerph-18-07949-s001.zip › ijerph-1266364-supplementary.pdf]

Table S1. Adsorption kinetic parameters for the biochar and magnetic biochar –Cd^{2+} adsorption system

Adsorbent	C_0 (mg/L)	Q_{exp} (mg/g)	Pseudo-first order			Pseudo-second order		
			Q_{cal}(mg/g)	K_1 (h ½)	R^2	Q_{cal} (mg g)	K_2 (g/mg.h)	R^2
Biochar	5	3	8.09	0.002	0.71	7.42	0.01070364	0.845
	10	5.9	11.25	0.0019	0.72	9.68	0.01033091	0.823
	20	9.81	15.98	0.003	0.67	13.35	0.01050249	0.865
	40	13.92	21.1 2	0.0028	0.65	16.2	0.01429242	0.925
	60	16.42	24.046	0.0033	0.77	18.12	0.0210552	0.95964
	80	18.15	28.22	0.0015	0.95	19.24	0.02203311	0.96326
	100	20.68	34.47	0.0019	0.92	21.1	0.02551189	0.97965
Magnetic Biochar	5	4.9	10.81	0.0033	0.81	6.9	0.0411065	0.8675
	10	8.83	15.49	0.0030	0.82	9.3	0.0425268	0.91368
	20	16.18	22.64	0.00471	0.85	18.6	0.0566124	0.8645
	40	23.88	27.66	0.00393	0.93	25.9	0.0632053	0.845
	60	29.4	34.10	0.002113	0.94	30.65	0.0752127	0.924
	80	42.86	47.94	0.0026933	0.96	43.3	0.0937112	0.968
	100	47.91	51.42	0.002296	0.98	48.4	0.1271858	0.98981

K_1(1/min): Rate constant of Lagergren equation and K_2 (mg/g min): Rate constant of Ho equation

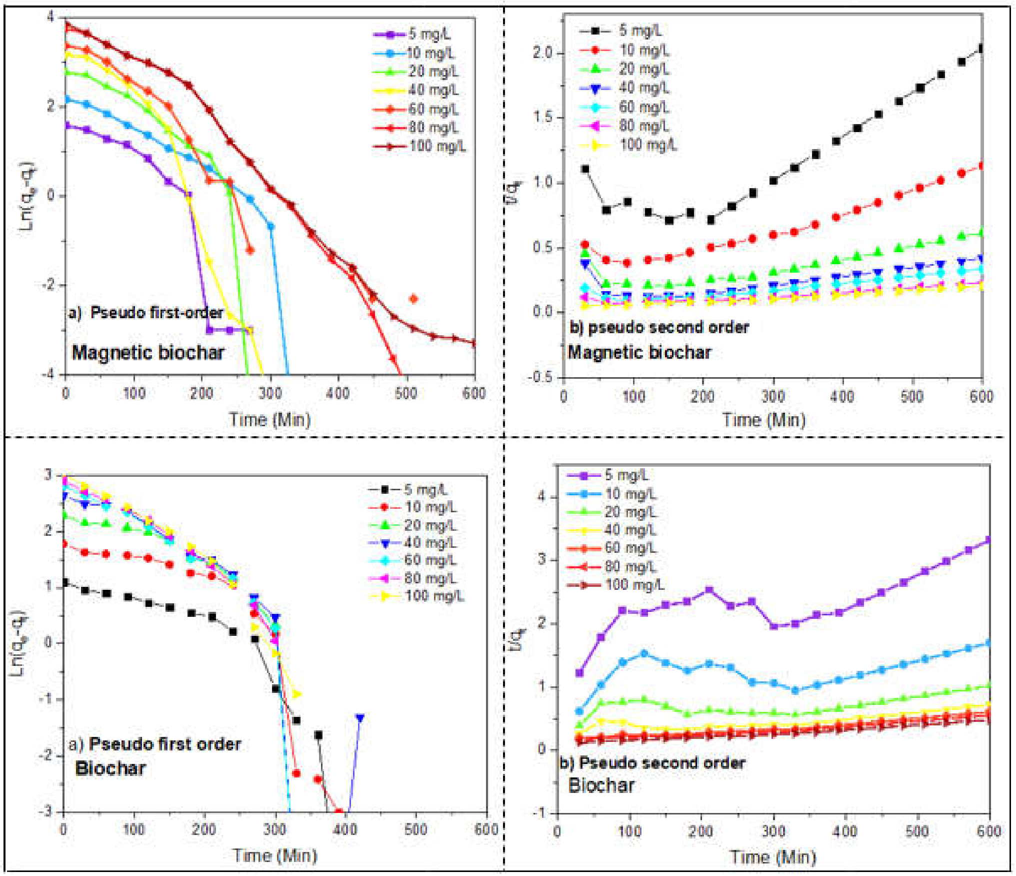

Figure S1. Kinetic models of the (a) pseudo-first order and (b) pseudo-second order

Table S2. Isotherm parameter of Cd^{2+} adsorption with biochar and magnetic biochar

Adsorbent	Langmuir			Freundlich			Temkin		
	Q_{max} (mg g^{-1})	K_L (L mg^{-1})	R^2	K_f (mL ^{3} g $^{-1}$)	1/n	R^2	K_T (L mg^{-1})	B (j mole $^{-1}$)	R^2
**Biochar**	18.38573	0.035196	0.96282	1.468859	0.79612	0.95313	0.294769	5.81032	0.77319
**Magnetic biochar**	43.95604	0.09798	0.99511	4.290126	0.62994	0.98311	1.003179	10.66556	0.91334

K_T: Temkin Constant, K_f: Freundlich Constant, K_L: Langmuir Constant and R^2: coefficient of determination

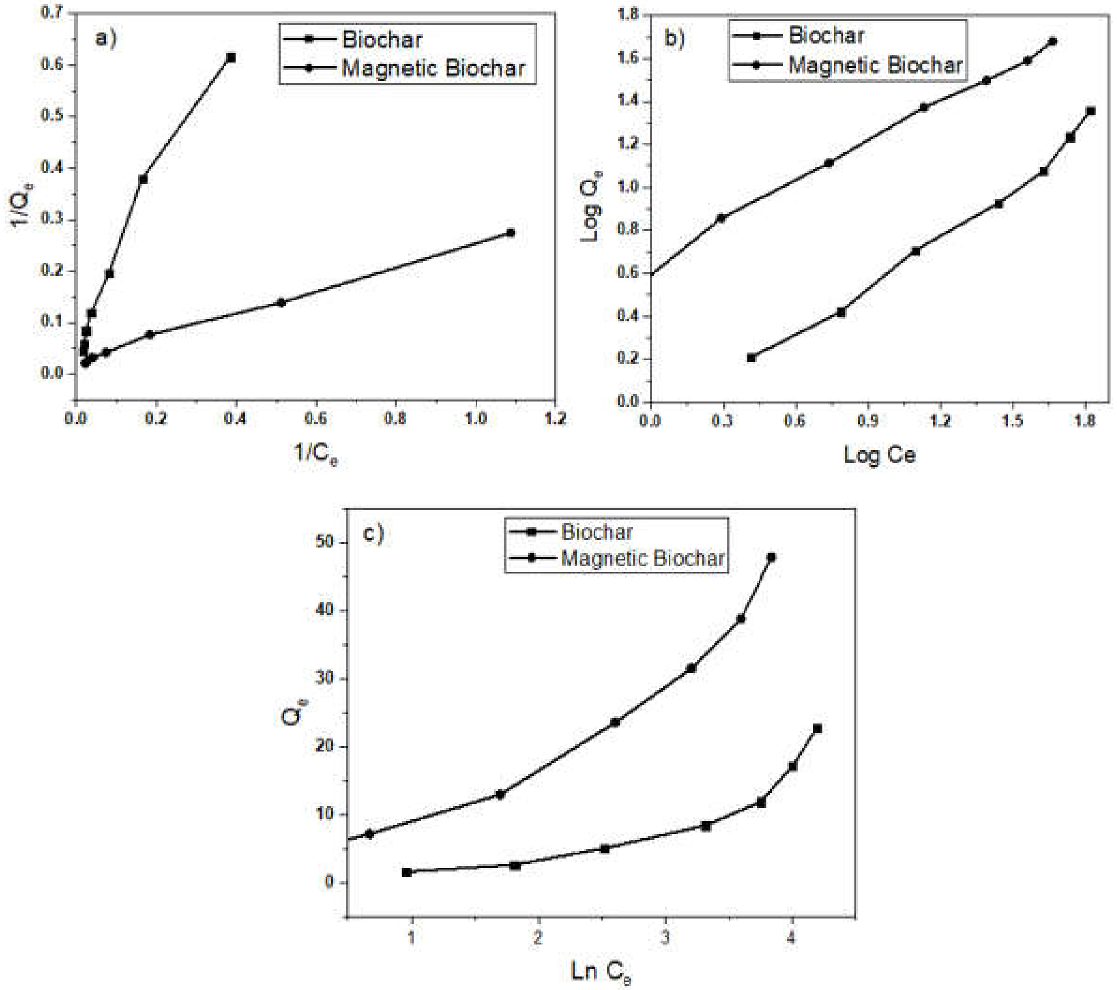

Figure S2. Fitting of isotherm models: (a) Langmuir isotherm, (b) Freundlich isotherm, and (c) Temkin isotherm

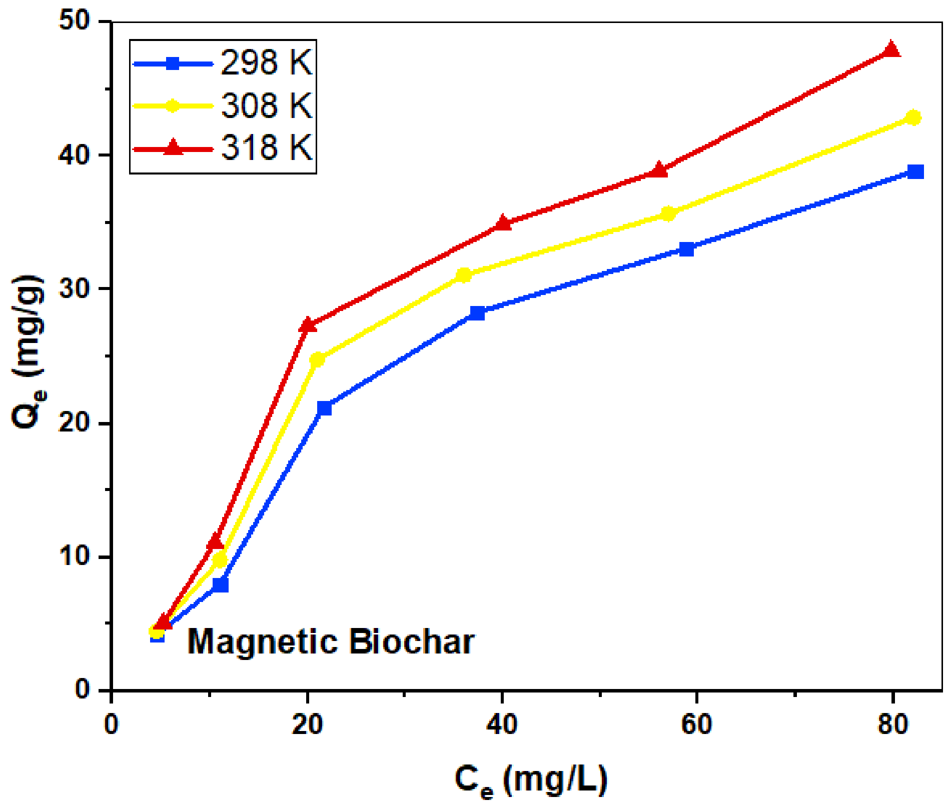

Figure S3. Influence of temperature on Cd^{2+} adsorption by magnetic biochar
